# Long-term mortality predictors of ICU fungaemia

**DOI:** 10.1017/S0950268821002235

**Published:** 2021-10-18

**Authors:** Peng Xie, Wenqiang Wang, Maolong Dong

**Affiliations:** 1 Department of Emergency Medicine, Nanfang Hospital, Southern Medical University, Guangzhou, Guangdong, China; 2 Department of Critical Care Medicine, Nanchong Central Hospital, the Second Clinical Medical College of North Sichuan Medical College, Nanchong, Sichuan, China; 3 School of Nursing, Chengdu Medical College, Chengdu, China; 4 Department of Burns, Nanfang Hospital, Southern Medical University, Guangzhou 510515, China

**Keywords:** Fungaemia, ICU, long-term, mortality predictors

## Abstract

Bloodstream fungal infections have a high mortality rate. There is little data about the long-term mortality rate of fungaemia.This study aimed to explore the mortality of fungaemia and the influencing factors associated with death. In total, 204 intensive care unit (ICU) patients with fungaemia from Multi-parameter Intelligent Monitoring in Intensive Care-III (MIMIC-III) Database were studied. Age, gender, major underlying diseases, data about vital signs and blood test results were analysed to identify the predictors of the mortality and prognosis of fungaemia in ICU patients. Cox regression models were constructed, together with Kaplan−Meier survival curves. The 30-day, 1-year, 2-year, 3-year and 4-year mortality rates were 41.2%, 62.3%, 68.1%, 72.5% and 75%, respectively. Age (*P* ＜ 0.001, OR = 1.530; *P* ＜ 0.001, OR = 1.485)，serum bilirubin (*P* = 0.016, OR = 2.125；*P* = 0.001, OR = 1.748) and international normalised ratio (INR) (*P* = 0.001, OR = 2.642; *P* ＜ 0.001 OR = 2.065) were predictors of both the 30-day and 4-year mortality rates. Renal failure (*P* = 0.009, OR = 1.643) performed good in prediction of the 4-year mortality. The mortality of fungaemia is high. Age，the serum bilirubin and INR are good predictors of the 30-day and 4-year mortality rates of fungaemia. Renal failure has good performance in predicting the long-term mortality.

## Introduction

Fungaemia has high short-term and long-term mortality rates. An Israel study conducted in the early 1990s reported an one-month mortality of 42% and a four-year mortality of 86% [1]. In recent decades, the incidence rate of invasive fungal infection has increased dramatically. Candidaemia is the fourth most common type of nosocomial blood stream infections (BSIs). The proportion of nosocomial BSIs induced by antibiotic-resistant organisms is increasing in US hospitals [2]. The increase in incidence is caused by advancement of modern medicine, such as organ transplantation [3, 4], broad-spectrum antimicrobial agents [5] and long-term central venous access devices [6], all of which can prolong the survival time while putting patients at high risk of fungal infections. There are many studies on bacterial and fungal BSIs [7, 8], however, only a few of them focus on predicting the long-term prognosis of fungaemia.

The number of antifungal treatment options has increased since the approval of caspofungin by Food and Drug Administration (FDA) in 2001 [9]. However, the long-term prognosis of fungaemia in ICU patients has been rarely reported. MIMIC-III Database was used in this study to explore good predictors of the mortality and prognosis of fungaemia in ICU patients.

## Materials and methods

This retrospective study was based on MIMIC-III Database [10]. The inclusion criteria were: (1) patients who had positive fungal blood culture test and received antifungal therapy and (2) patients who were over 18 years old. The exclusion criteria were patients with incomplete data. This large open database consists of about 53 000 ICU adult patients' de-identified health-related data from 2001 to 2012 at Beth Israel Deaconess Medical Center in Boston, MA. It was jointly established by Massachusetts Institute of Technology, Phillips Healthcare and Beth Israel Deaconess Medical Center. Access to and use of it were granted after ‘Human Research (Curriculum Group)’, a course of the Collaborative Institutional Training Initiative (CITI) Program, was finished (Certification Number: 29641289).

The variables extracted from MIMIC-III Database included age on the day of blood culture test, gender and major underlying diseases, such as diabetes mellitus(diabetes), congestive heart failure, chronic obstructive pulmonary disease (COPD), renal failure, liver disease and solid tumours. The variables of vital signs were mean blood pressure (mean bp, mmHg) on the day of blood culture test. Other variables of blood test results were serum bilirubin level (bilirubin, mg/dl), serum creatinine level (creatinine, mg/dl), blood urea nitrogen (BUN, mg/dl), INR and pulse oxygen saturation(SPO_2_）on the day of blood culture test. Severity of fungaemia was estimated using the sequential organ failure assessment (SOFA) score on the day of blood culture test. The patients'comorbidity scores were evaluated with the Elixhauser_vanwalraven scoring system [11]. The worst value was taken when a variable was repeatedly tested.

### Statistical analysis

The count data were represented by case number (*n*), and the intergroup differences were analysed with Chi-square test or Fisher's exact test. The Shapiro−Wilk normality test showed that some data did not conform to a normal distribution (*P* < 0.05). Therefore, the measurement data in this study were described by P50 (P25, P75) and a non-parametric rank-sum test was used for intergroup comparison. The factors associated both 30-days and the 4-year mortality were identified. The factors whose *P* values were ⩽0.1 in univariate analyses were entered into Cox regression model in the way of forward selection (conditional LR) until the final model was achieved. Kaplan–Meier survival curves for significant predictors of the 4-year mortality were constructed and analysed using the log-rank test. All analyses were performed using SPSS (IBM SPSS Statistics 25).

## Results

**Fig. 1.**
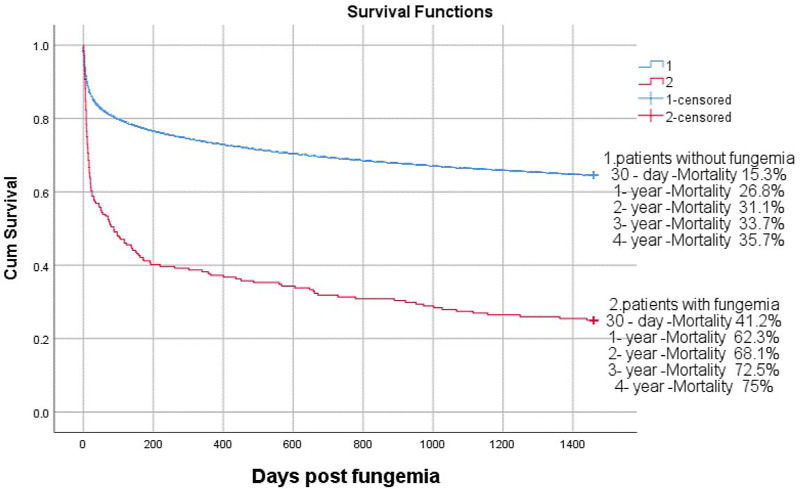
Overall survival.

Among the 38 512 ICU patients aged 18 years or more registered in the database, 204(0.53%) with fungaemia who met the inclusion criteria were analysed, and their 225 fungaemia episodes were reviewed. The patients with fungaemia had a median age of 64.7 years (interquartile range: 51.4–74.6) and 57.8% of them were male. The 30-day and 4-year mortality rates were 41.2% and 75%, respectively, while the rest of the population of the database were 13.6% and 34.7%, respectively, as shown in Figure 1. The median age of the 38 315 patients over 18 years old and without fungaemia was 65.9 years (interquartile range: 52.7–78.1) and 56.6% of them were male. The fungi isolated are listed in Table 1.

**Table 1.** Common fungus isolated

Fungi	Number isolated(%)
*Candida albicans*	80(35.5)
Candida (Torulopsis) glabrata	67(29.7)
Candida parapsilosis	30(13.3)
Candida tropicalis	17(7.5)
Candida dubliensis	8(3.5)
Yeast	7(3.1)
Candida krusei	4(1.7)
Candida lusitaniae	4(1.7)
Candida kefyr	3(1.3)
Candida guilliermondii	3(1.3)
Crptococcus neoformans	1(0.4)
Saccharomyces cerevisiae	1(0.4)

### Thirty-day mortality and risk factors

Table 2 shows the univariate and Cox regression predictors of mortality by 30 days. Age, SOFA score, Elixhauser_vanwalraven, bilirubin and INR were important predictors of 30-day mortality. The decrease of meanbp was also a good predictor of 30-day mortality. The 30-day mortality rate of patients with liver disease or steroid therapy also increased. The 30-day mortality rate of patients with solid tumours decreased. It was not affected by factors such as the choice of antifungal treatment and the type and number of fungi.

**Table 2.** Predictors of 30-day mortality at the time of fungaemia

	Univariate analysis			Cox regression	
Variable	Survived (*n* = 120)	Unsurvive (*n* = 84)	*P* value	*P* value	OR (95% CI)
Female	49 (40.8%）	37 (44.0%）	0.668		
Age (years)	62.5 (50.6, 72.1)	67.6 (55.8, 76.9)	0.033	＜0.001	1.530(1.277−1.907)
SOFA score	3 (0, 7)	7 (3, 12)	0.000	0.001	3.330(1.675−6.622)
Elixhauser_vanwalraven	8 (3, 12)	7 (13, 18)	0.002	0.019	1.726(1.096−2.719)
Underlying conditions					
Diabetes	29 (24.2%）	30 (35.7%）	0.085		
Congestive heart failure	28 (23.3%）	32 (38.1%）	0.029		
COPD	25 (20.8%）	14 (16.7%）	0.476		
Renal failure	18 (15.0%）	26 (31.0%）	0.009		
Liver disease	25(20.8%）	38 (45.2%）	0.000	0.030	1.781(1.059−2.998)
Solid tumour	8 (6.7%）	1 (1.2%）	0.084	0.041	0.125(0.017−0.922)
Previous treatment					
Immunosuppressant	12(10.0%）	10(11.9%）	0.655		
Steroid therapy	54 (45.0%）	57(67.9%）	0.002	0.001	2.005(1.245−3.229)
Renal replacement therapy	4 (3.3%）	10 (13.1%）	0.013		
Caspofungin	37(30.8%）	26(31.0%）	1.000		
Fluconazole	51(42.5%）	34(40.5%）	0.885		
Micafungin	27(22.5%）	22(26.2%）	0.618		
Other antifungal drugs	5(4.2%）	2(2.4%）	0.702		
Monitoring value					
Bilirubin (mg/dl)	0.7 (0.4, 1.9)	1.8 (0.7, 7.4)	0.000	0.016	2.125(1.148−3.934)
Creatinine (mg/dl)	1.3 (0.9, 2.1)	2.1 (1.1, 3.5)	0.002		
INR	1.3 (1.2, 1.8)	1.9 (1.4, 2.6)	0.000	0.001	2.359(1.433−3.882)
Bun(mg/dl)	35 (19.2, 55)	53.5 (37.5, 85)	0.000		
Meanbp(mmHg)	58 (51, 66)	55.3 (43.2, 60)	0.022	0.001	2.642(1.466−4.762)
SPO_2_	94 (91, 96)	92 (89, 94)	0.026		
Type of fungus					
*C. albicans*	50 (41.7%）	27 (32.1%）	0.188		
*C. tropicalis*	6 (5.0%）	11 (13.1%）	0.107		
*C. glabrata*	35 (29.2%）	23 (27.4%）	0.875		
*C. parapsilosis*	17 (14.2%）	11 (13.1%）	1.000		
Other spp.	12 (10.0%）	12 (14.3%）	0.382		

### Four-year mortality and survival curves

**Fig. 2.**
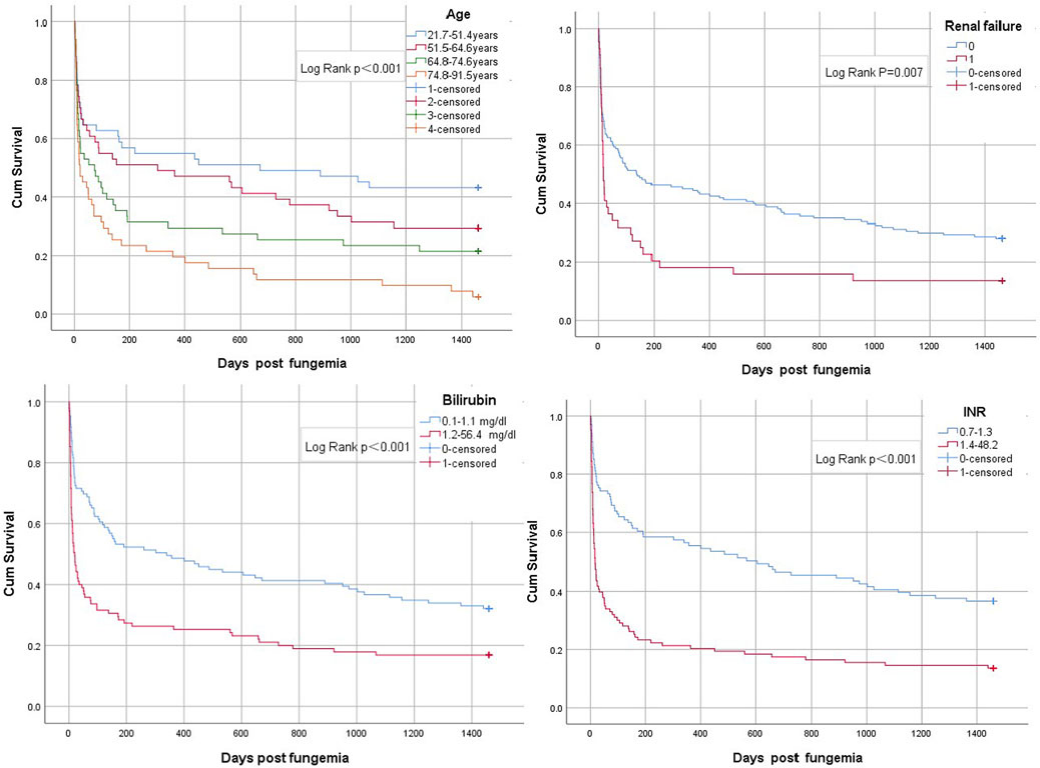
Kaplan–Meier survival curves of age, renal failure, bilirubin and INR.

As shown in Table 3，In univariate analyses, increased mortality was associated with age, SOFA score, Elixhauser_vanwalraven, congestive heart failure, renal replacement therapy，bilirubin，INR，Bun and Meanbp. In Cox regression analysis, the age, bilirubin，renal failure and INR were accurate in predicting the 4-year mortality. Kaplan−Meier survival curves for quartiles of age and binary variables of renal failure, bilirubin, INR are shown in Figure 2.

**Table 3.** 4-year mortality at the time of fungaemia

	Univariate analysis			Cox regression	
Variable	Survived (*n* = 51)	unsurvive (*n* = 154)	*P* value	*P* value	OR (95% CI)
Female	23 (45.1%）	63 (41.2%）	0.668		
Age (years)	52.5 (44, 65.1)	67.5 (55.3, 76.8)	0.000	＜0.001	1.485(1.274−1.731)
SOFA score	2 (0, 7)	6 (1, 9)	0.005		
Elixhauser_vanwalraven	6 (3, 12)	11 (5, 16)	0.007		
Underlying conditions					
Diabetes	10 (19.6）	49 (32.0%）	0.109		
Congestive heart failure	8 (15.7%）	52 (34.0%）	0.013		
COPD	11 (21.6%）	28 (18.37%）	0.681		
Renal failure	6 (11.8%）	38 (24.8%）	0.052	0.009	1.643(1.130−2.389)
Liver disease	12(23.5%）	51 (33.3%）	0.223		
Solid tumour	2 (3.9%）	7 (4.6%）	1.000		
Previous treatment					
Immunosuppressant	7(13.7%）	15(9.8%）	0.440		
Steroid therapy	24 (47.1%）	87(56.9%）	0.257		
Renal replacement therapy	3 (5.9%）	12 (7.8%）	0.013		
Caspofungin	11(21.6%）	52(34.0%）	0.116		
Fluconazole	22(43.1%）	63(41.2%）	0.870		
Micafungin	15(29.4%）	34(22.2%）	0.345		
Other antifungal drugs	3(5.9%）	4(2.6%）	0.702		
Monitoring value					
Bilirubin(mg/dl)	0.6 (0.3, 1.9)	1.3 (0.4, 4.6)	0.029	0.001	1.748(1.245−2.252)
Creatinine(mg/dl)	1.2 (0.8, 2.1)	1.7 (1.0, 2.9)	0.055		
INR	1.3 (1.2, 1.5)	1.6 (1.3, 2.3)	4E-06	＜0.001	2.065(1.471−2.898)
Bun(mg/dl)	29 (15, 54)	56 (28, 72)	0.002		
Meanbp(mmHg)	59 (52, 67)	56 (45.5, 62.8)	0.038		
SPO_2_	94 (91, 95)	93 (90, 96)	0.262		
Type of fungus					
*C. albicans*	18 (35.3%）	59 (38.6%）	0.740		
*C. tropicalis*	3 (5.9%）	14 (9.2%）	0.571		
*C. glabrata*	14 (27.5%）	44 (28.8%）	1.000		
*C. parapsilosis*	10 (19.6%）	18 (11.8%）	0.165		
Other spp.	6 (11.8%）	18 (11.8%）	1.000		
Number of isolate fungus					
1 fungus	46(90.2%）	138(90.2%）	1.000		
2 fungi	5(9.8%）	14(9.2%）	1.000		
3 fungi	0(0%）	1(1.2%）	1.000		

## Discussion

In this study, results show that age，INR and bilirubin are good predictors of short-term and long-term mortality of fungaemia. In particular, the INR is statistically significant in predicting 30-day mortality (*P* = 0.001, OR = 2.359 (1.433–3.882)), but also in predicting 4-year mortality (*P* ＜0.001, OR = 2.065(1.471–2.898)). Therefore, INR is a good predictor of mortality in fungaemia. In other reports, INR was associated with in-hospital mortality [12, 13]. One possible explanation we have found is the systemic inflammation that often occurs in patients with fungaemia and the interference between inflammatory pathways and the coagulation system. This may lead to organ dysfunction, such as an increase in bilirubin. Deteriorated organ function leads to increased short-term and long-term mortality in patients with fungaemia.

Bilirubin can be used as an independent predictor of the short-term and long-term prognosis of fungaemia. Bilirubin is the principal tetrapyrrole, bile pigment and catabolite of haem [14]. It inhibited LPS-induced B-cell proliferation and cytokine production from splenic macrophages to prove immunosuppression associated with hyperbilirubinaemia reported by Nazir M Khan [15] *et al*. Pierrakos *et al*. [16] found that hyperbilirubinaemia was independently associated with increased mortality in critically ill patients. At the same time, one research study also showed that purification therapy was effective in reducing bilirubin levels [17]. It is debatable whether the short-term and long-term prognosis of mycosis can be improved by lowering bilirubin levels.

Bilirubin, as a biomarker for the prevalence of chronic diseases and prediction of all-cause mortality [18], is also a new biomarker for successful ageing [14]. The short-term and long-term mortality rates increased significantly with age, three observational studies had similar results [1, 19, 20]. During the 4-year follow-up period, the mortality rate increased steadily with time compared with other ICU patients. Further research is needed to study this issue and how to reduce the mortality of fungaemia among young people. Ribero *et al*. [20] reported that age was also an important prognostic factor for melanoma, and that increase in age led to decrease in survival rate. The Elixhauser_vanwalraven system [11] was used to score the patients' underlying diseases and to analyse and predict the mortality of fungaemia and SOFA score reflects the acute status of organ functions. This study shows that the Elixhauser_vanwalraven score and the SOFA score is a good predictor of the 30-day mortality of mycosis.

Renal failure could be used as an independent predictor of the long-term prognosis of fungaemia. Similar results have been found in other disease studies, such as Toshitaka Okabe [21] *et al*.'s study, which found that renal failure was associated with cardiovascular death in patients with heart failure. This is because fungaemia aggravates chronic inflammation of the kidney, which increases the risk of death [22].

In this study, nine cases of patients with fungaemia accompanied by solid tumour, only one died within 30 days. The 30-day mortality rate of fungaemia patients with solid tumour was reduced. Chen [23] et al. reported that there was no difference in 30-day mortality of patients with mycosis accompanied by solid tumour. The possible explanation was that the data are too small, resulting in selective errors.

In addition, we have illustrated that the 1-month, 1-year, 2-year, 3-year and 4-year mortality rates of fungaemia are 41.2%, 62.3%, 68.1%, 72.5% and 75.0% among ICU patients in this study, respectively. Siriluck Anunnatsiri *et al*. [19] reported that the overall in-hospital mortality of fungaemia was 56.1% at Srinagarind Hospital in northeastern Thailand. Lillie *et al*. [7] reported that the 30-day mortality and the 3-year mortality of BSIs were 15% and 48.75%, respectively. Fungaemia and bacteraemia have completely different short-term and long-term prognosis.

Among the fungal strains in this study, there is a case of fungaemia caused by *Saccharomyces cerevisiae* infection. General patient information is, male, 50.8 years old, with renal failure and liver disease, long-term use of glucocorticoids and immunosuppressants. After diagnosis of fungaemia, the survival period was 218 days. *S. cerevisiae* is widely used in baking and brewing industry, which is a very rare infection in human beings. Patricia Muñoz [24] suggested that the use of *S. cerevisiae* should be carefully reassessed, especially in immunosuppressed or critically ill patients. Davide Fiore Bavaro reported that targeted therapy might enhance or inhibit immunity in patients with autoimmune idiopathic diseases. However, the drugs would have some adverse reactions, such as opportunistic infections [25].

This study has six limitations: ① the sample size was small because the data were from a single centre and the incidence of fungaemia was low, which might lead to non-universal findings. ② Fungaemia are often ‘opportunistic’ infections, caused by important antibiotic selective pressure, parenteral nutrition and major abdominal surgery. However, this study is a retrospective study rather than a prospective study, which inevitably leads to the limitation. ③ There were no data control group in this study. ④ The study did not stratify outcomes according to therapeutic interventions. ⑤ Polymicrobic infections were not used as a prediction; ⑥ The long-term and short-term mortality of fungaemia might be different in different areas because of differences in healthcare quality, economic development and culture.

## Conclusion

In conclusion, the incidence rate of fungaemia is 0.53% among ICU patients. There is an appreciable and gradual increase in mortality after an episode of bloodstream fungal infection. The SOFA score and Elixhauser vanwalraven score have good performance in predicting the 30-day mortality. Treatment of organ dysfunction and control of underlying diseases may help improve the short-term prognosis of fungaemia. Age，INR and bilirubin are good predictors of the short-term and long-term mortality rates of fungaemia. Renal failure has good performance in predicting the long-term mortality.

## Data Availability

The data that support the findings of this study are from database. This large open database consists of about 53 000 ICU adult patients' de-identified health-related data from 2001 to 2012 at Beth Israel Deaconess Medical Center in Boston, MA. It was jointly established by Massachusetts Institute of Technology, Phillips Healthcare and Beth Israel Deaconess Medical Center. Access to and use of it were granted after ‘Human Research (Curriculum Group)’, a course of the Collaborative Institutional Training Initiative (CITI) Program, was finished (Certification Number: 29641289).
